# Fediša Modikologo: breaking the intergenerational cycle of violence against women and children. Theoretical framework and protocol for a prospective cohort study

**DOI:** 10.12688/wellcomeopenres.23513.2

**Published:** 2025-08-07

**Authors:** Rachel Jewkes, Leane Ramsoomar, Jani Nothling, Samantha Willan, Venice Mbowane, Esnat Chirwa, Shibe Mhlongo, Maureen Phakoe, Desiree Pass, Amanda Zembe, Louis Sibiya, Ishen Seocharan, Charntel Paile, Laura Washington, Nataly Woollett, Bianca Dekel, Nwabisa Jama-Shai, Mercilene Machisa, Pinky Mahlangu, Boitumelo Seepamore, Nicola Christofides, Tracy Glass, Darshini Govindasamy, Stanley Carries, Asiphe Ketelo, Naeemah Abrahams

**Affiliations:** 1 Gender and Health Research Unit, South African Medical Research Council, Pretoria, Gauteng, 0002, South Africa; 2 Office of the Executive Scientist, South African Medical Research Council, Pretoria, Gauteng, 0002, South Africa; 3 School of Public Health, University of the Witwatersrand, Johannesburg, Gauteng, South Africa; 4 School of Health Systems and Public Health, University of Pretoria, Pretoria, South Africa; 5 School of Social Applied Human Sciences (Social Work), University of KwaZulu-Natal, Durban, KwaZulu-Natal, South Africa; 6 Biostatistics Unit, South African Medical Research Council, Pretoria, Gauteng, 0002, South Africa; 7 Project Empower, Diakonia Centre, Durban, KwaZulu-Natal, South Africa; 8 School of Nursing and Public Health, University of KwaZulu-Natal, Durban, South Africa; 9 Burden of Disease Research Unit, South African Medical Research Council, Cape Town, South Africa; 10 Health Systems Research Unit, South African Medical Research Council, Durban, KwaZulul-Natal, South Africa; 11 School of Public Health and Family Medicine, University of Cape Town, Cape Town, South Africa

**Keywords:** Violence against women; violence against children; femicide; cohort; mental health; recovery; resilience; risk factors;

## Abstract

In South Africa, after two decades of national femicide surveillance, we know comparatively little about what places women who experience intimate partner violence (IPV) at risk of intimate partner femicide. Further we have not mapped the multi-generational health, social and economic impact of severe IPV on women subjected to it, and their children, nor the consequences of help-seeking, nor described what helps, STET recovery trajectories. This study aims to deepen understanding of risk factors for femicide and the health, social and economic impacts of severe IPV on women and their families, including understanding risk and resilience to intergenerational cycling of violence. It further aims to describe how statutory and community measures operate to enable recovery and safety. Following pilot research, we developed a prospective questionnaire-based cohort study with three components, and plan for nested qualitative research. The primary cohort will enrol 12,000 women experiencing severe IPV, recruited using non-probabilistic methods (mostly referral from services and community members, and chain-recruitment). Following a baseline interview, participants will complete annual on-line surveys to track key outcomes for five years. The main questionnaire will measure exposure to range of different forms of IPV in the past year, lifetime trauma exposure history, childhood background, health, social and economic circumstances and help-seeking practices. A sub-cohort of the women (a 20% sub-sample), will be followed more intensively over 3 years. Among these, the children aged 6 years and over, of consenting mothers, will also be followed for three years. Deaths in the cohorts will be tracked through the National Population Register through participants’ national identity numbers. Mixed-methods verbal autopsies will be conducted with friends or family members of deceased participants. Results will guide femicide prevention nationally, and will build understanding of what is needed to prevent intergenerational cycling of violence and enable recovery of exposed women and children.

## Plain language summary

‘Fediša Modikologo: breaking the intergenerational cycle of violence against women and children. Theoretical framework and protocol for a prospective cohort study’ describes the plans for research that will be conducted by the South African Medical Research Council and partners in four diverse sites in South Africa with women who have experienced severe intimate partner violence and their children. This study aims to help us to understand what places women at risk of the most severe impacts of intimate partner violence, including loss of life, and to describe the myriad of ways in which their experiences, and attempts to protect themselves and their families, impact their health, social circumstances and finances. It also aims to help us understand how children are impacted by growing up in a home with violence and what may influence whether they go on to use violence themselves in their lives. We are recruiting 12,000 women who have recently experienced severe intimate partner violence into the study and we will interview them with a questionnaire, and provide counselling, over 5 years. We will as k a sub-group of these women to come for more intensive interviews, and if they agree, to invite their children, aged 6 years and over, as well. We will identify and investigate any deaths. The results will guide femicide prevention nationally, and will build understanding of what is needed to prevent the worst outcomes and enable recovery among exposed women and children.

## Introduction

Intimate partners, i.e. current or former husbands or boyfriends, perpetrate more than half of the female murders in South Africa, a country where deaths from interpersonal violence are ranked second among the risk factors for the burden of disease in women ^1, 2^. Intimate partner femicide (IPF) is the most extreme consequence of intimate partner violence (IPV), which usually precedes the fatal event ^3^. For the last two decades, IPF surveillance has been conducted through dedicated surveys by Abrahams and colleagues ^2^. This has revealed a substantial decline in the IPF rate from 1999 – 2009, from a high starting rate, but little improvement thereafter ^2^. South Africa has the highest IPF rate of any country with data ^2^. Most recent estimates show that the age-standardised rate was 4.9 per 100,000 female population in 2017 ^2^. Global estimates are available for femicide by an intimate partner or family-related killings, with a global prevalence of 1.1/100,000 female population ^4^. The countries reporting the highest rates being Surinam (4.3/100,000 female population) and Belize (2.1/100,000 female population) ^4^.

South Africa does not have surveillance of the prevalence of IPV. The 2016 South African Demographic and Health Survey reported that 21.5% of women aged 18–49 years had experienced physical or sexual IPV in their lifetime, and 10.3 % in the past year ^5^. The Sub-Saharan African regional average for these prevalences is about two-fold higher than these figures, which does not align with what is known about the very high rate of IPF ^6^. It is recognised that IPV can be substantially under-reported in surveys, and that attention to methodology and interview context are crucial for good data, but reported prevalence is also influenced by a loyalty of many women who experience IPV towards their abuser, and the pressures they may experience to stay, or leave, these relationships from family and communities. This is much less often explored in South African research on IPV. Thus, although drivers of IPV are relatively well-established at a global level ^7, 8^, there are many gaps in our understanding of the relative importance and mechanisms of actions of risk factors, as well as the microdynamics that contribute to the risk of severe IPV. A consequence this is that knowledge of which women are most at risk of attempted or actual IPF is not available. Additionally, there is insufficient information on the relative effectiveness of available sources of support for women experiencing IPV.

To (effectively) provide assistance for women experiencing IPV, we need more understanding of the impact of severe IPV exposure on women, and the reasons why women stay in violent relationships. These factors generally are rooted in patriarchal gender norms and span economic, socio-cultural and psychological concerns, including, among others, receiving economic support from their partner, a desire to keep the family together, not having natal family support, barriers created by the psychological distress of experiencing severe IPV, fearing stigma and having altered cognitive schemas related to abusive relationships (e.g. Stockholm Syndrome) ^9– 13^. The relative importance of these is not well understood and has not been studied in South Africa, or a country with a similar socio-cultural context.

It is well-recognised that children who live in households where there is domestic violence frequently witness this, and are often also subjected to other forms of violence, and are consequentially at higher risk of perpetrating or experiencing IPV ^7^. This is known as the intergenerational cycle of violence (IGCV). Although the cycle has been long recognised, and the risk pathways are known to be multiple, their relative importance, as well as resilience factors, require further research. Postulated pathways include social learning of the use of violence, and the impact of abuse and violence exposure on neurocognitive development, resulting in lower self-esteem, empathy and remorse, and more instrumental behaviour, as well as a greater propensity for mental ill-health, borderline personality, harmful alcohol use and substance use ^14– 21^. It has also been postulated that these factors are aggravated by harsher and neglectful parenting, in the face of IPV ^22^. Our aim is to deepen our understanding of the impact of IPV on South African children and adolescents, and the drivers of IGCV, so that we are better able to develop interventions to reduce the harm experienced by affected children and adolescents and ultimately to break this pernicious cycle.

## Aims

The overall aim of the Fediša Modikologo Study is to deepen understanding of risk for IPF, deepen understanding of the myriad of impacts of severe IPV on women and their families, and especially the impact of IPV exposure on children and adolescents, and to understand more about what assistance women exposed to severe IPV need and what more effectively enables their safety. The specific aims are to enable description of:

1. the risk and protective factors for, and pathways to, IPF and attempted femicide
2. women’s social, economic and health trajectories and pathways to recovery when experiencing severe IPV, and the impact of help-seeking,
3. the extent to which available assistance enables recovery after severe IPV and protects women from further IPV
4. the impact of severe IPV exposure on children and adolescents’ health and developmental outcomes, and the risk and protective factors for, and drivers of, intergenerational cycling of violence

## Theoretical framework for the study

**Figure 1.**
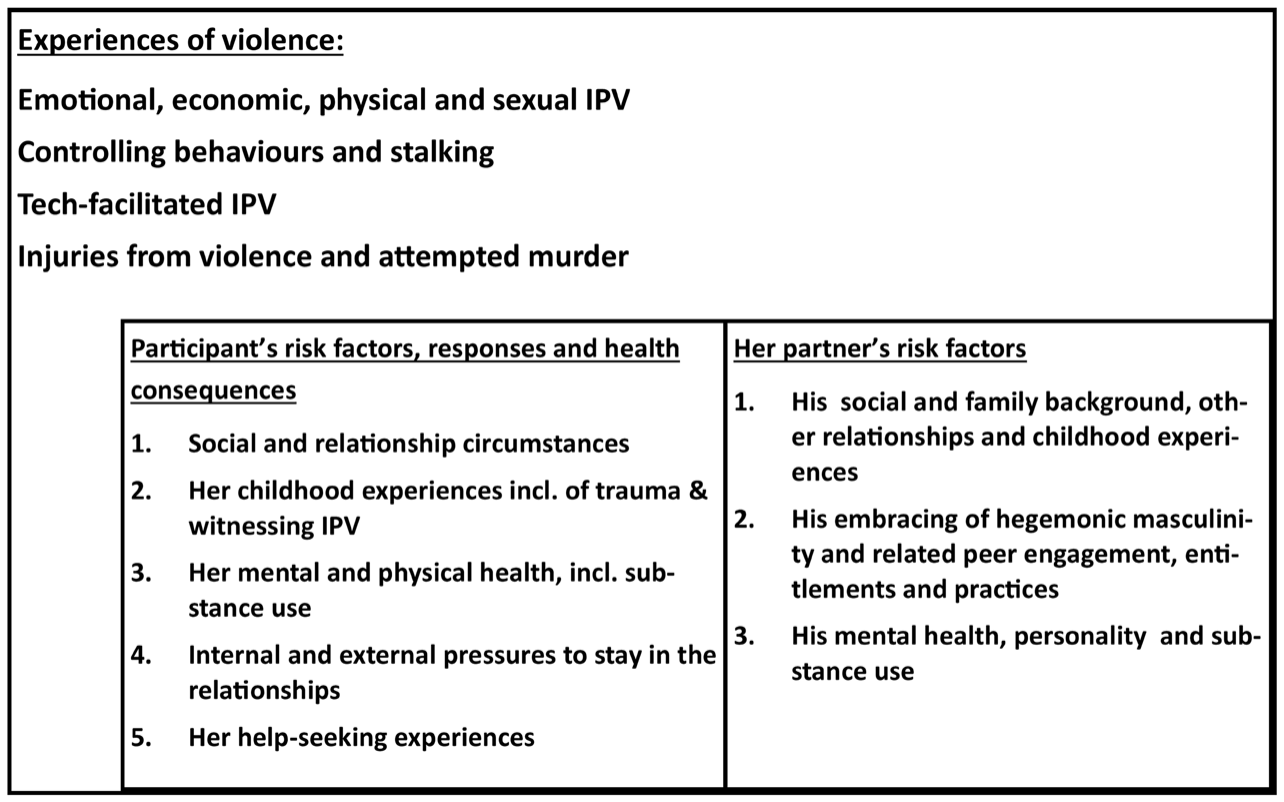
Theoretical framework for the main cohort (Cohort A).

The aims will be researched through a study with three nested cohorts, and associated other research. They are viewed as nested as the first cohort in Cohort A, Cohort B is a sub-set of Cohort A’s members, and Cohort C will be children of some of the Cohort B mothers. Figure 1 presents the theoretical framework for the main cohort in this study (Cohort A). We hypothesise that a woman’s risk of femicide, or attempted femicide, is a product of the violent and controlling practices that she experiences from her male partner in the relationship. It will be further influenced by a range of forces that stem from her background and socio-economic circumstances, influenced by her experiences of trauma, violence and key relationships in her childhood, including witnessing abuse of women at home. Her mental and physical health, resilience and coping, including substance misuse, will be influenced by her experiences of violence, and these will also impact her response in the face of further IPV. Her risk will also be influenced by her partner’s social and economic circumstances, the degree to which he embraced ideals of hegemonic masculinity with the associated entitlements, peer influences, and norms related to the use of violence, as well as by his personality, mental health and substance use. These will also be shaped by his exposure to violence in childhood and witnessing abuse. We hypothesise that how she responds, for example by leaving or staying, will influence her subsequent IPV exposure. Her continuing risk of IPV will also be related to her experiences in help-seeking for relationship problems and the internal and external pressure that she encounters to stay or leave the relationship, and whether she has children. As we are not interviewing her partner, we are only able to collect such information as we can through her reports. This limits our ability to understand more about his background and childhood and we have found most women do not know very much.

**Figure 2.**
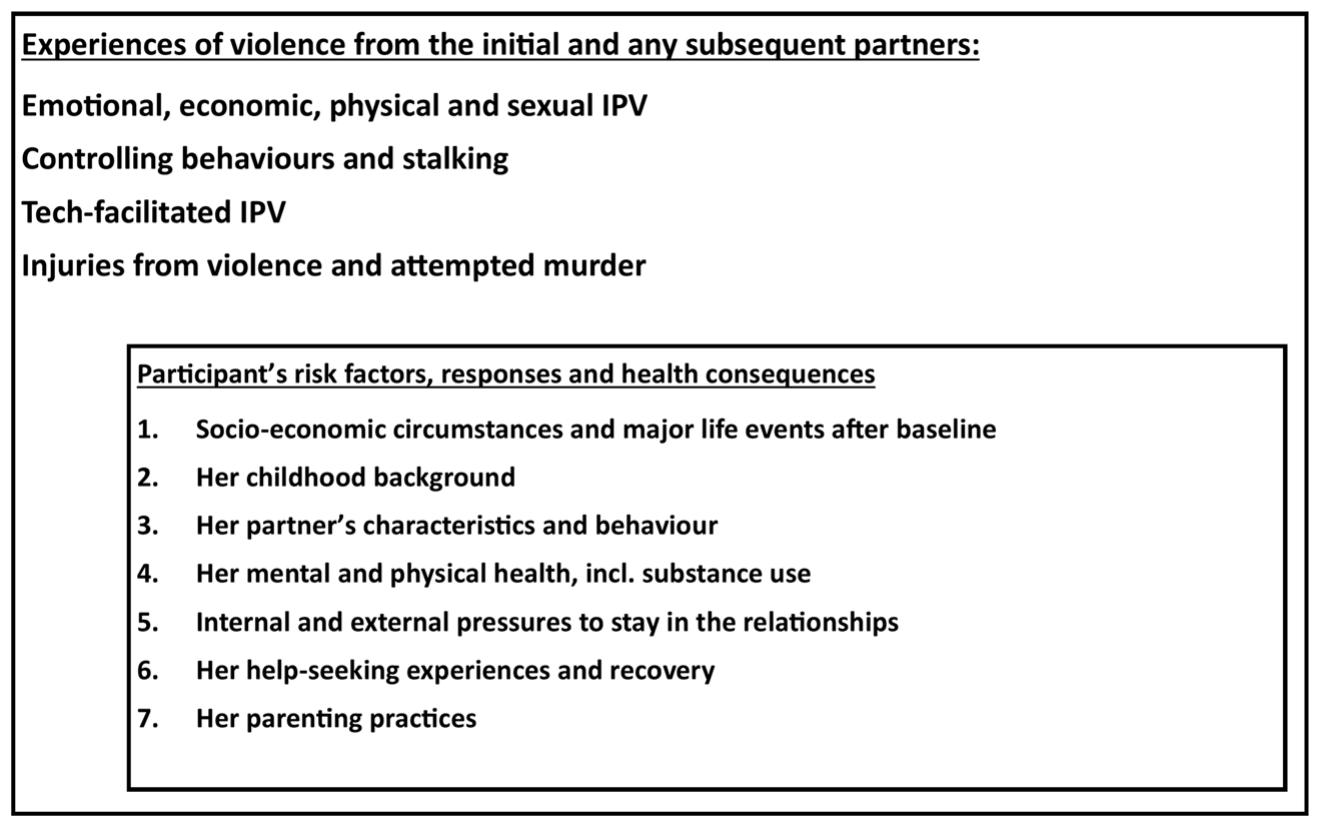
Theoretical framework for the first sub-cohort (Cohort B).

In order to achieve the second aim, a sub-cohort of women from the cohort A will be followed intensively to study impact of severe IPV on their and their families’ lives (Cohort B). This cohort will enable research into the trajectory of, and factors influencing, further IPV and injuries experienced by the women, her socio-economic situation and major life events, her parenting practices, her physical and mental health and substance use, evidence of recovery, internal and external pressures to stay in a violent relationship and her experiences with help-seeking and leaving ( Figure 2). It is designed so that the longitudinal data can be analysed to reveal different patterns among the variables (using dummy variables with binary, ordinal or categorical outcomes and exposures, and latent class analysis) and determining the factors associated with different trajectories and outcomes.

**Figure 3.**
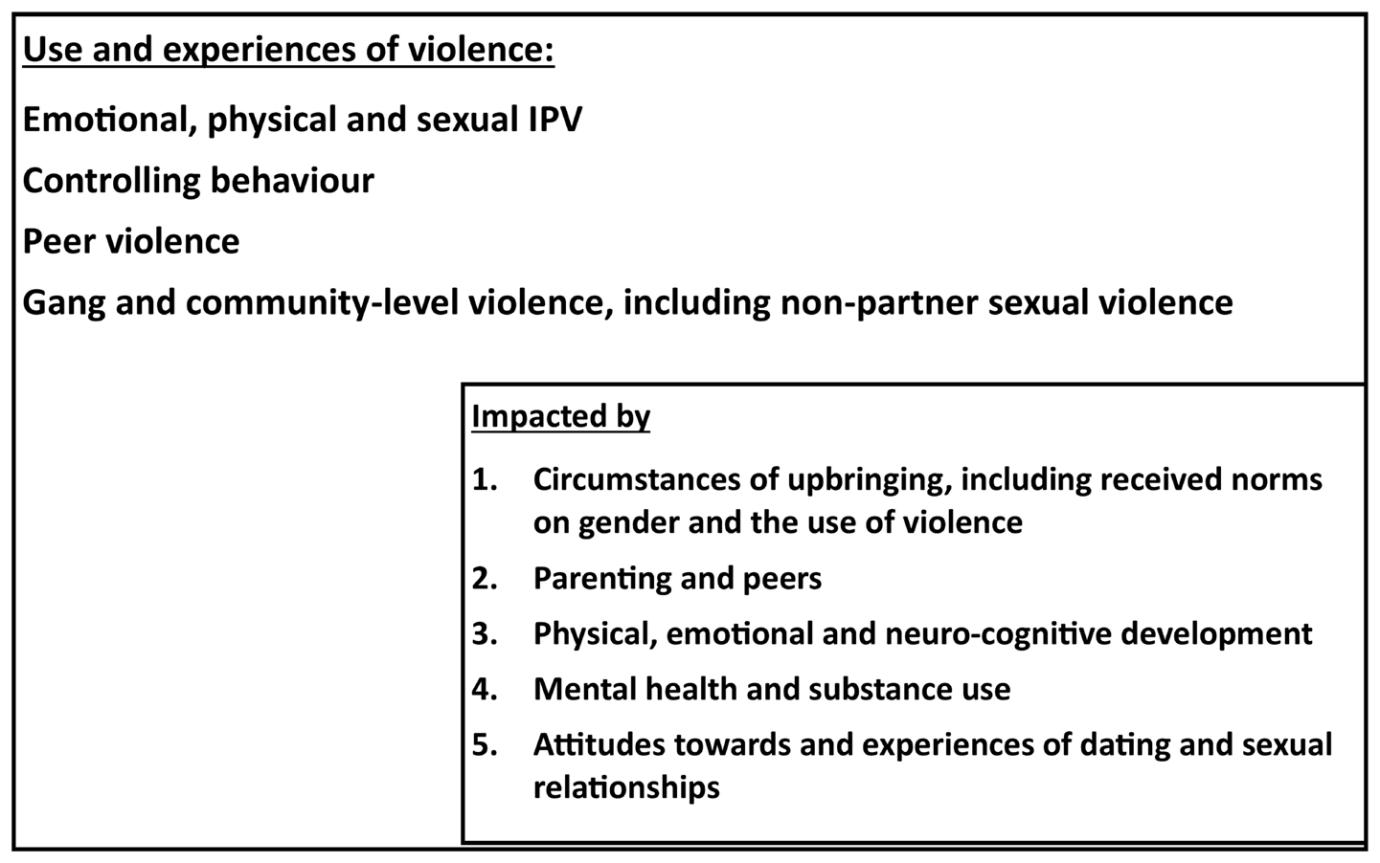
Theoretical framework for the second sub-cohort (Cohort C).

To accomplish the third aim, we will have a second sub-cohort (Cohort C), consisting of female and male children of the Cohort B mothers. The data from the cohort will enable us to describe the impact of growing up in a home with IPV exposure on children and adolescents’ health and developmental pathways, and deepen understanding of IGCV. The cohort is designed to collect data on the impact on children’s mental health, exposure to childhood trauma and other violence, socialization with peers, and their school achievement and development longitudinally. Figure 3 presents a theoretical framework underpinning this cohort. We hypothesise that the social and economic context at home; parental availability, mental health and substance use, and parenting practices; norms on gender and the use of violence; the use of violence at home and exposure to violence; all impact children and ultimately their propensity to use violence, and vulnerability to violence. Further they impact their physical and neurocognitive development, early experiences of violence, children’s own mental health and substance use, and their attitudes towards and exposure to dating and sex.

## Settings

This research is being conducted across four sites in South Africa. Two are in major urban areas, in the cities of Pretoria and Durban (Tshwane and eThekwini Municipalities in Gauteng and KwaZulu-Natal). One is in a small rural town, Modimolle, in Waterberg District of Limpopo Province. The final site is in Stellenbosch in the Cape Winelands District of the Western Cape. The catchment area for the latter two sites are both rural farming and small town communities. The sites are in four of the country’s nine Provinces and have been selected to ensure that all of South Africa’s population groups, defined in the Apartheid era, but enduringly lines of social and economic division of society, can be recruited into the study. They are also positioned to enable participants speaking almost all of South Africa’s 12 official languages to be recruited into the study. In each location, study offices have been established.

## Participants

Eligible participants in the main cohort, are aged 18–45 years and women, either identified at birth or self-identifying. In the latter case, living as a woman for at least a year, irrespective of hormone treatment or gender-reassignment surgery. They must have a current or ex- male partner who subjects them to severe IPV. In the case of an ex-partner, the relationship should have ended in the 12 months prior to the first interview, and he should still be feared, intimidating, stalking or contacting her in a way she finds threatening. The operational definition of severe IPV is that she would have experienced severe physical IPV (i.e. having been hit with a fist, or something that could hurt, strangled, kicked, dragged, beaten up or threatened with/injured by a weapon), been raped or forced into sex, or engage in sexual acts, against her wishes, or have had threats to kill her, threats to harm any children in order to hurt her, stalking, or she should fear him. Alternatively, she would have experienced multiple forms of severe controlling behaviour, such as being confined to the home, prevented from working or earning income, or experienced jealous surveillance. The definition of severe IPV was developed in order to recruit participants at higher risk of intimate partner femicide. Eligible participants must be able to communicate in any of the study’s six languages (English, isiZulu, SePedi, isiXhosa, Setswana, and Afrikaans) and have a South African identity number, to allow tracking of mortality in the cohort in the National Population Register.

## Participant recruitment

**Figure 4.**
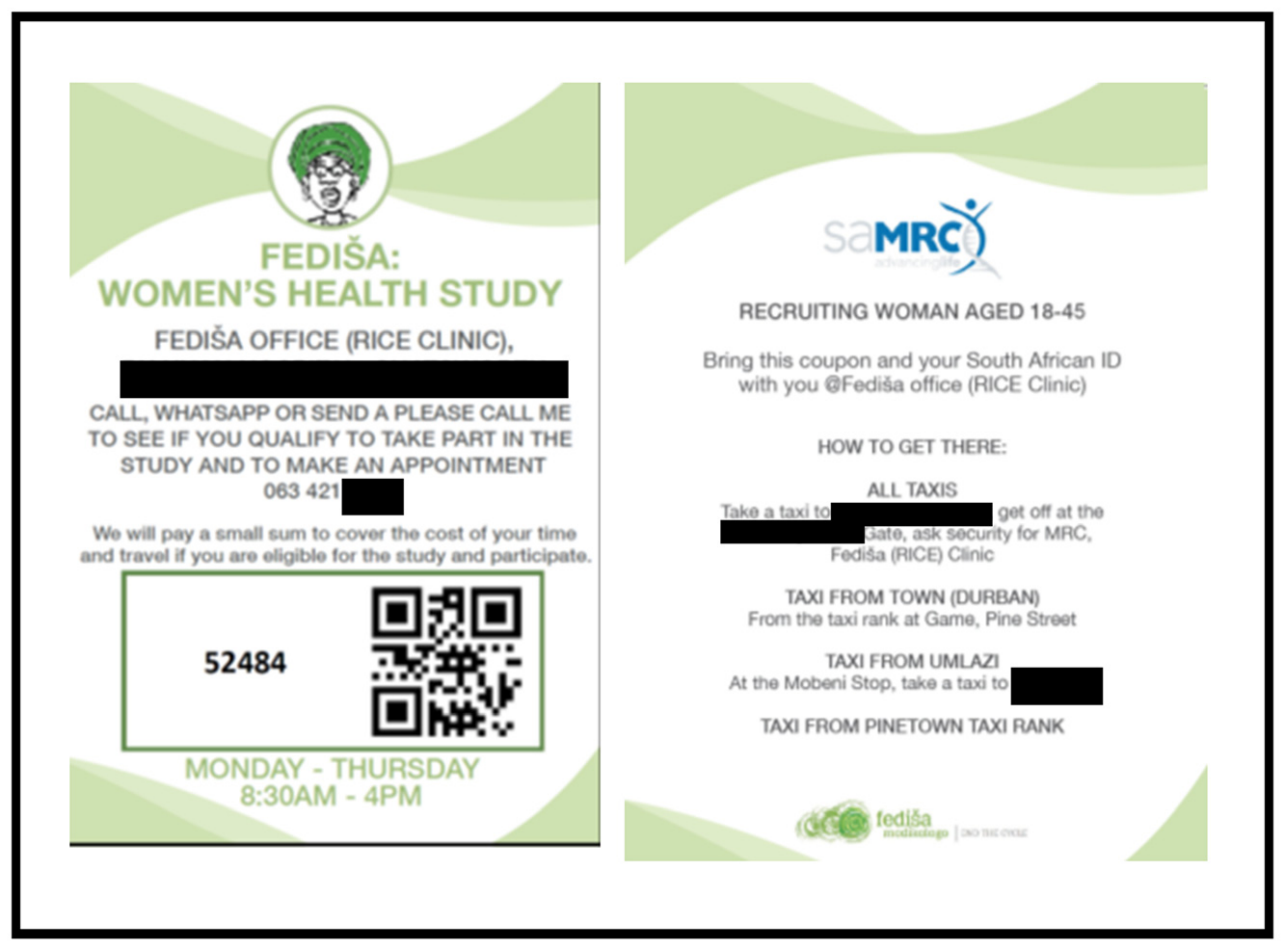
Coupons used in chain referral recruitment.

There are two main routes for participant recruitment. The first is referral from agencies and individuals in the vicinity of the study sites who have cause to engage with, or come to know about, women experiencing severe IPV. These include non-governmental organisations and government services providing counselling and support services, including gender-based violence (GBV) organisations and community workers, Victim Empowerment Projects, social workers, the police and courts providing Protection Orders (POs). The second is participant chain referral (a variation on respondent driven sampling). In this case, when a participant has been interviewed, she is asked if she knows another woman in the same situation as she is in and is given a leaflet about the study and a coupon to hand to the other woman. Up to three coupons are distributed per interviewee. The coupons invite a person to call the office to check eligibility for the study (see Figure 4). When someone does so, a brief screen for eligibility is conducted on the phone and, depending on the outcome, an interview date is scheduled.

Participants for the first sub-cohort (Cohort B) are to be a sub-sample of those interviewed in the main cohort who agreed to further interviews, more easily can access the study site and have their own telephone or SIM card (the last criterion is to ensure safety and confidentiality for the women). In each office, we will identify a sub-group of participants who meet these criteria, examine the ages of co-residing children and invite participants who agreed to participate in further research. We will select those with co-residing children aged 6 years and over, who agree for their children to participate in the research, initially, so that we can meet our recruitment target of 600 adult women enrolled per office, and 600 children (300 boys and 300 girls), of which 450 would be aged 13 and over and 150 aged 6–12 years. The children will be invited to agree to participate, after their mothers have provided informed consent. Much of our decision-making will be driven by the need to meet these criteria, but where we have choice due to a larger number of potential participants than required, we will randomly select among them.

## Sample size

The minimum sample size for the cohort was guided by the expected prevalence of women who experience severe IPV in the population and our estimates of mortality rates among women experiencing severe IPV. Based on the 2016 South Africa Demographic and Health Survey, we assumed that nationally there is an annual prevalence of severe IPV experience of 3.6% and a total population of women aged 18 years or older for 2022 of about 17 million, the latter based on estimates from Thembisa 4.4 model ( https://www.thembisa.org/content/downloadPage/Thembisa4_4report). From this we estimated that about 600,000 women nationally experience severe IPV per annum. We further assumed that there are 1027 IPF cases per annum, based on previous study estimates ( https://doi.org/10.1371/journal.pmed.1004330). Assuming these cases of IPF come from women who had experienced severe IPV, we calculated the risk of IPF among severe IPV of 1/556. A more cautious minimum cohort size was then calculated with a risk of 1/650, based on observing at least 80 IPF cases over a minimum follow-up period of 5 years. The minimum cohort size of 12,000 was considered sufficient at varying IPF risk for a minimum of 80 cases over 5 years (see Table 1).

**Table 1.** Cohort sizes for varying IPF risk.

Risk of IPF	cohort size	# of IPF cases/ annum	total IPF cases in 5 yrs	IPF/Others † ratio
1/650	12000	16	78	0.007
	14000	18	91	0.007
1/556	10000	20	100	0.010
	12000	24	100	0.010
1/400	10000	25	125	0.013
	12000	300	150	0.013
1/300	10000	33	165	0.017
	12000	40	198	0.017

Others†: Total of other women i.e. those surviving and those killed by a person who is not their intimate partner

We examined whether a minimum cohort size of 12,000 would have sufficient power to compare well known risk factors of IPF such as partner’s heavy drinking. Table 2 shows the varying statistical power for a cohort size of 12,000, assuming several scenarios of IPF risk that would give at least 80 IPF cases over 5 years of follow up. The power to detect varying prevalence risk factors for a minimum cohort size of 12000 seemed sufficient. However, we are mindful that all estimates depend on the assumptions about the risk of IPF among women experiencing severe IPV.

**Table 2.** Power to detect varying prevalence of risk factors at assumed incidence of IPF.

Risk of IPF=1/650; nratio=0.007				Risk of IPF=1/556; nratio=0.010				Risk of IPF=1/400; nratio=0.013				Risk of IPF=1/300; nratio=0.017			
Power	OR	p1	p2	Power	OR	p1	p2	Power	OR	p1	p2	Power	OR	p1	p2
0.48	1.52	0.35	0.45	0.62	1.52	0.35	0.45	0.72	1.52	0.35	0.45	0.83	1.52	0.35	0.45
0.51	1.56	0.3	0.4	0.64	1.56	0.3	0.4	0.75	1.56	0.3	0.4	0.85	1.56	0.3	0.4
0.55	1.62	0.25	0.35	0.69	1.62	0.25	0.35	0.79	1.62	0.25	0.35	0.88	1.62	0.25	0.35
0.80	1.86	0.35	0.5	0.92	1.86	0.35	0.5	0.97	1.86	0.35	0.5	0.99	1.86	0.35	0.5
0.82	1.91	0.3	0.45	0.93	1.91	0.3	0.45	0.97	1.91	0.3	0.45	0.99	1.91	0.3	0.45
0.85	2.00	0.25	0.4	0.94	2.00	0.25	0.4	0.98	2.00	0.25	0.4	0.99	2.00	0.25	0.4
0.97	2.33	0.3	0.5	0.99	2.33	0.3	0.5	1.00	2.33	0.3	0.5	1.00	2.33	0.3	0.5
0.97	2.46	0.25	0.45	1.00	2.46	0.25	0.45	1.00	2.46	0.25	0.45	1.00	2.46	0.25	0.45

p1=prob of exposure (partner has drinking problem) among the controls; p2=prob of exposure (partner has drinking problem) among the controls. OR= Odds ratio; nratio= cases/controls

The sample size of the two sub-cohorts was based on pragmatic considerations, including available resources. We considered that a 20% sub-sample of the main cohort (n=2400 women) would be more than sufficient to enable sub-group analysis, and analyses where the sample was divided such as using latent class analysis techniques. We anticipate a high retention rate as enrolment in Cohort B is an indication of being willing to participate in further interviews (as all participants have already been interviewed).

## Measures

Table 3 summarises the main constructs and measures used in the main cohort. Where possible we have used established measures, that were locally-developed, or tested and validated, in South Africa. However, for many of the constructs there are no established measures, so we designed measures for our purposes and contexts. We developed, or adapted, many of the measures after cognitive testing and exploratory qualitative research, and formal piloting, with review of their psychometric properties.

**Table 3.** Main constructs and measures used in the main cohort.

	# items	Details of measure	Source
**Socio-demographic characteristics**			
Age, education, racial group, relationship status, religiosity	17		developed for the study
Children's ages & residence	11		developed for the study
Food insecurity & shocks	4	Frequency of lack of food at home in the last 4 weeks. Three items asking about having no food at home, going hungry overnight and having no food for a day and a night. Response options: often, sometimes, rarely, never	Household Hunger Scale of Deitchler M *et al*. ^23^
Social grants	3		developed for the study
Occupation	1		developed for the study
Income	3		developed for the study
Maintenance	6	Frequency and quantity of money & groceries received, and Court ordered maintenance	developed for the study
Home, car and goods ownership	11		developed for the study
Social support	7	Social support from friends and family who she can count on, share joys and problems including relationship problems with. Response on 4 point Likert scale	Social Support Appraisals Scale adapted from Vaux *et al*. ^24^
Community connectedness	4	Typical item: 'If there was a fist fight in this area would people do something to stop it?' 4-level categorical responses	Adapted from Jewkes *et al*. ^25^
**Her childhood**			
Parents & childhood caregivers and relationship quality	11	Items asking about who raised her and 3 items asking about her mother insulting her, being overly controlling and unpredictable when growing up. 4-point Likert response scale.	Three items from Measure of Parenting Scale (MOPS) , Parker G *et al*. ^26^
Childhood poverty	7	Frequency of hunger and lack of money in childhood, type of area raised in, home ownership and moving between homes	Adapted from the Household Hunger Scale of Deitchler M *et al*. ^23^
Relationship with her mother now	5	Items comparing her relationship with her mother now and when she was a child, 4 items asking about her mother seeing her as a disappointment, not behaving as she should, being very critical and uninterested. 4-point Likert response scale.	Developed after formative research
Childhood trauma	14	Items ask bout experiences of physical abuse, emotional abuse and neglect, physical hardship and sexual abuse. Never, sometimes, often and very often response options.	Childhood Trauma Questionnaire, Bernstein *et al*. ^27^
Witnessing IPV	14	Items ask about acts of emotional & physical IPV and being severely injured or killed. Items refer to mother and 'another woman at home'. Responses asking about whether this was seen, heard, told about or didn't happen.	Developed after formative research using a modified CTS style
Bullying/being bullied	5	Items about verbal abuse, social isolation, physical abuse and damage to property. 4-point response categories from never to very often	Items from Mynard H *et al*. ^28^
Forced first sex and non-partner rape	6	Questions ask about being forced into sex by a man who was not her husband or a boyfriend. Items ask about ever and in past year, being forced by two or more men, being drink or drugged when forced, and forced first sex. 4-point response options capture frequency. Also age first time and forced first sex.	Adapted from Jewkes *et al*. ^29^
Gender norms	9	Typical item: "A woman should show respect for her husband by doing what he tells her to do." 4-point Likert scale responses.	Adapted from Jewkes *et al*. ^30^, Pulerwitz & Barker ^31^ after formative research
**Her main partner**			
Socio-demographics and employment	13	Age, race, country of birth, education, employment, low income stress, relative income, and household contributions	developed for the study
His children & treatment of children at home	4	Whether he has children with other mothers, punishing children, children fearing him and her fear he may hurt the children	developed for the study
Other partner/wife	3	Another wife or partner and whether there is a new relationship	developed for the study
Arrests, prison, fights with men and weapons	6	Items with yes/no/don't know response options asking about arrests, having been in prison, fighting with other man and having weapons	variables developed for Jewkes *et al*. ^32^
Mental health	9	Items ask about the last two weeks and whether her partner was depressed, had lost interest in things, having sleep problems, restless and worrying; one edge and fearful; and hypervigilant and having reimagining episodes.	developed for the study by adapting DSM-5 cross-cutting symptoms for mood and anxiety disorders.
Drugs and alcohol	7	Items on drinking alcohol, frequency of drunkenness, arguments when drunk, whether she or any children are afraid when he is drunk, drug use and irritability when unable to get drugs	developed for the study
Machiavellian egocentricity	5	Measure of anti-social personality disorder. Typical item: "__(name)_________ sometimes lies just to see if he can get someone to believe him". 4-level Likert response options	Sub-scales of the Psychopathic Personality Inventory- Revised (PPI-I) ^33^
Blame externalisation	5	Measure of anti-social personality disorder. Typical item: 'When things go wrong for __(name)_________ its always someone else's fault". 4-level Likert response options	Adapted sub-scale of the Psychopathic Personality Inventory- Revised (PPI-I) ^33^
Splitting	4	Items cover aspects of binary thinking characteristic of splitting. Typical item: "__(name)_________ tends to think people are all good or all bad". 4-level Likert response options	developed for the study
Second partner: demographics, violence & weapons	11	Items drawn from the above questions	developed for the study
**Her relationship and IPV**			
Controlling behaviour	9	Items measure aspects of controlling behaviour in relationships Typical item: "__(name)_________ hasn’t let me wear certain things". 4-point responses from never true to very true.	Adapted from Jewkes *et al*. ^34^
Paranoid jealousy	22	Items measure symptoms of paranoia including mistrust, hypervigilance, difficulty with forgiveness, defensive attitude in response to imagined criticism, preoccupation with hidden motives, fear of being tricked. Typical item: "__(name)_________ asks his friends to check on my behaviour and report to him". 4-point responses from never true to very true.	Developed for the study after formative research drawing on definition of paranoia from: https://mhanational.org/conditions/paranoia-and-delusional-disorders
Stalking	2	Two items asking about having ever been stalked and whether others had been used to harass her. 4-point frequency response categories	Adapted from Tjaden & Thoennes ^35^
Quarrelling	1	Single item on frequency of quarrelling, with response options from never to everyday	developed for the study
Emotional IPV	10	Items on being belittled or humiliated, appearance insulted, scared, threatened, his hurting others to get at her, and open infidelity ever and in the past year. 4-point Likert scale responses	Adapted from WHO questionnaire after formative research ^36^
Economic IPV	10	Items on refusing to earn, stopping her earning, taking her earnings, not giving money for the home, squandering money, monitoring her account, or taking her possessions. In the past year or ever. 4-point Likert scale responses	Adapted from WHO questionnaire ^36^ after formative research
Porn and filming	6	Items on frequency of forced exposure to pornography, making unwanted sex videos, demanding or sending sexual photographs, and distributing these on-line in or before the past year.	developed for the study after formative research
Social media & phone control	8	Items on frequency of use of social media, humiliating her or hacking her accounts on social media and contacting her under false pretences in or before the past year. Also using her phone to control and track her with an app, pin drops or otherwise checking her phone. 4-point response categories.	developed for the study after formative research
Physical IPV	8	Items on the frequency of slapping, hair pulling, pushing, shoving, burning, choking, kicking, dragging, beating up, strangling, and threats or injury with a weapon. 4-point response categories.	Adapted from WHO questionnaire ^36^
Sexual IPV	6	Items on the frequency of being forced into sex physically, agreeing from fear, being forced into humiliating or degrading sexual acts, or strangulation during sex ever and in the past year. 4-point response categories.	Adapted from WHO questionnaire (Garcia-Moreno 2005) ^36^
IPV pregnancy	5	Items on pregnancy and whether she was beaten in pregnancy and lost a pregnancy due to violence. Binary response options.	Adapted from WHO questionnaire ^36^
Attempted femicide and fear	6	Items on being afraid of her partner, fearing or worrying that he may kill her, attempts to kill her, or suspicion of an attempt, and details of what happened (open question).	Developed for the study after formative research
Injuries & traumatic head injury	16	Items on the number of times injured, types of injury, and body parts injured, loss of consciousness (head injury or strangulation), being dazed or confused and loss of memory from head injuries.	Adapted from WHO questionnaire ^36^ and traumatic brain injury questions from Setnik 2007 ^37^
Medical treatment	7	Items on needing medical attention (whether or not accessed) after injuries, hospital admissions and length of stay in public or private hospitals.	developed for the study
Stockholm syndrome	19	Items framed around core cognitive distortions, psychological damage & love-dependency. 4-point Likert response options.	Adapted from DLR Graham *et al*. ^10^
**Her health and substance use**			
Physical health	1	Single item asking whether medication is taken for a range of conditions	developed for the study
Gad-7 - anxiety	7	Generalised anxiety disorder measure	Spitzer RL, *et al*. ^38^
AUDIT	10	Harmful alcohol consumption measure	Saunders JB *et al*. ^39^
Drug use	3	Drugs ever taken, when last taken and what is taken	developed for the study
PHQ-9- depression	9	Depression in last 2 weeks	Kroenke K *et al*. ^40^
Suicide	4	Suicidal thoughts in the past month and week, suicidal plans and previous attempts	
Borderline	17	Nine core items, modified to remove double-barrelled questions for ease of completion. Mostly binary responses	https://111.wales.nhs.uk/borderlinepersonalitydisorder/
Lifetime trauma	13	Lifetime trauma experiences, modified for South Africa. Listed witnessed traumatic events with binary responses. Questions which affected them the most.	Modified life events checklist ^41^
PTSD	18	Items measuring PTSD and complex PTSD	International Trauma Questionnaire, Cloitre M *et al*. ^42^
Internal & external IPV stigma	11	Items on: Being insulted, blamed, shamed, humiliated & gossiped about due to IPV, and on shame, self-blame and social withdrawal due to leaving the relationship. 4-level response categories asking about frequency.	Modified from sex worker stigma items Jewkes *et al*. ^43^; Milovanovic *et al*. ^44^ after formative research
Her use of violence	7	Yelled, insulted or humiliated him, threw things, hit, slap, or beat, threaten with or use of a weapon, ever and past year. 4-level frequency response categories	Modified CTS design, developed after formative research
**Leaving and seeking help**			
Leaving the relationship & returning	20	Items about contemplating leaving, reasons for having not done it, reasons for returning after leaving. Mostly binary response categories	developed after formative research
Stigma of leaving, and positive feelings after leaving	15	Items on: Being insulted, blamed, shamed, humiliated & gossiped about due to leaving the relationship, and on shame, self-blame and social withdrawal due to leaving the relationship. 4-level response categories asking about frequency. As well as three items on personal strengthen gained from leaving.	developed after formative research, modified from IPV stigma questions stem; positive items modified from positive views on sex work Jewkes *et al.*, Milovanovic *et al*. ^43, 44^
Sources of help and assessment of value	64	16 items on persons spoken to; items on to whom reports were made, what was done (mostly binary responses) and how useful it was (scale 1-10): priest (2), police (8), social worker (6), NGO (8), helpline (2), Protection Order (22)	developed after formative research
Capabilities	7	5 items of ICECAP-A with added variables on feeling respected and physically safe; 4 level response categories.	ICECAP-A scale Al-Janabi H *et al*. ^45^ adapted for South Africa

## Qualitative research and pilot testing

To cognitively test measures and better understand constructs prior to measures development, we conducted qualitative research in the three sites that were staffed at this phase of the study. In each, we used stakeholder networks to identify women who had experienced severe IPV and who were willing to participate. We recruited 42 participants (13 in Stellenbosch (Afrikaans and isiXhosa-speaking), 14 in Durban (isiZulu and isiXhosa-speaking) and 15 in Pretoria (Setswana and SePedi-speaking)), and enrolled them in panels that met weekly over eight weeks to reflect on eight different sets of measures and share their experiences. The sessions were 2–3 hours long, and each was facilitated by two researchers, in the study languages. Data were recorded by note-taking and audio recording. The main areas covered were: well-being measures; economic and emotional IPV and controlling behaviour; why women stay with violent partners including IPV and related stigma; causes of conflict in relationships, and circumstances leading up to IPV; when women use violence; measuring borderline personality; norms on gender and use of violence; emotional exhaustion and feeling stressed; experiences of leaving violent relationships and help-seeking; Stockholm syndrome; parenting and attachment measures; witnessing IPV and community violence in childhood; the role of alcohol and other substance use in IPV; and what questions women can answer about their male partners.

After this we developed the questionnaires for testing in the main pilot study. We fielded two long questionnaires, to enable more measures to be included so that we could assess psychometric properties of measures, test overall performance, and reduce items and measures. The pilot study was conducted in April and May 2024, in the four study sites. Each site had an office and a team of interviewers and we followed the procedures for the main study in all respects with use of REDCap ^46^ for interviews, and use of chain-referral recruitment. We enrolled 470 participants in total, 236 participants completed one questionnaire and 234 completed the other. Once the data were completed we reviewed responses, considered response prevalence, variability and correlations, and examined reliability, factor loading, and uniqueness to make decisions about reducing measures and items.

After we developed the Cohort B questionnaire, we conducted pilot interviews testing new measures with 330 participants from Cohort A who were asked to complete some further questions. The data were analysed as per the Cohort A pilot study. We did not test the whole questionnaire as many of the items were lightly edited to ask ‘since the last interview…’, but in substance the same as those used in Cohort A.

## Other qualitative research

The study has several different types of qualitative data. There are data from the measures development phase of Cohort A, which included discussion of certain issues, with probing and cognitive testing of the questionnaire, which also entailed some broader discussion of the topics. The same approach is planned for Cohort C and will generate similarly rich data. We also have a open-ended questions embedded in the REDCap questionnaire, and a comment log in REDCap which enables comments with further information to be added after any of the questions. This has provided an additional dimension to understanding women’s experiences that normally would not be available. In addition to this we are planning a range of nested qualitative research projects. These are being planned with interviews to be conducted with women experiencing severe IPV and service providers, NGOs and social workers, providing assistance to them. 

## Outcomes

For the main cohort, there are two primary outcomes. First, IPF, defined as the murder of a woman by an intimate partner (i.e. a current or ex-husband/boyfriend, same-sex partner or a rejected would-be lover). In South Africa, the term ‘murder’ refers in law to ‘intentional and unlawful killing’. The second is attempted femicide (murder), including acts and injuries that could have resulted in loss of life. This is derived from a combination of direct reports of attempted murder or suspicious situations that could have resulted in loss of life were the participants believed her partner was behind them (e.g. breaks ‘failing’ on a car after being parked at home), as well as loss of consciousness from head injury or strangulation, reports of strangulation, burns, gunshots, stabbing to the neck, torso or genitals, or suffocation ^47^. Secondary outcomes relate to women’s experience of further IPV, mental health and psychological well-being. For the first sub-cohort, the primary outcomes related to further experience and intensity of severe IPV, experience of injuries, socio-economic circumstances, and physical and mental health. For the children’s sub-cohort, the primary outcomes relate to mental health and social well-being and experience or perpetration of violence.

## Developing REDCap and language translation

REDCap was used to develop and customise multilingual data collection by integrating the Multi-Language Management (MLM) module. MLM is a REDCap feature that facilitates the setup and translation of questions into multiple languages, ensuring that participants can complete the surveys in their preferred language. To maintain data consistency, a thorough language validation process was implemented, with translations reviewed by native speakers in the research team, who understood the context, to confirm that the meaning of the questions remained consistent across languages. This step was crucial in ensuring that participants from diverse linguistic backgrounds provided accurate and comparable responses, ultimately improving the quality of data collected.

The REDCap system also incorporated advanced data validation tools and automated checks to ensure accuracy during data entry. Branching logic was employed to create personalized survey paths, ensuring that participants answered only relevant questions based on their responses. For example, certain questions could be skipped or additional ones prompted, reducing respondent fatigue and increasing the precision of data collection. These features, combined with REDCap’s built-in validation checks, minimized errors during data entry and ensured that the information collected was accurate and reliable across different languages.

## Procedure

**Figure 5.**
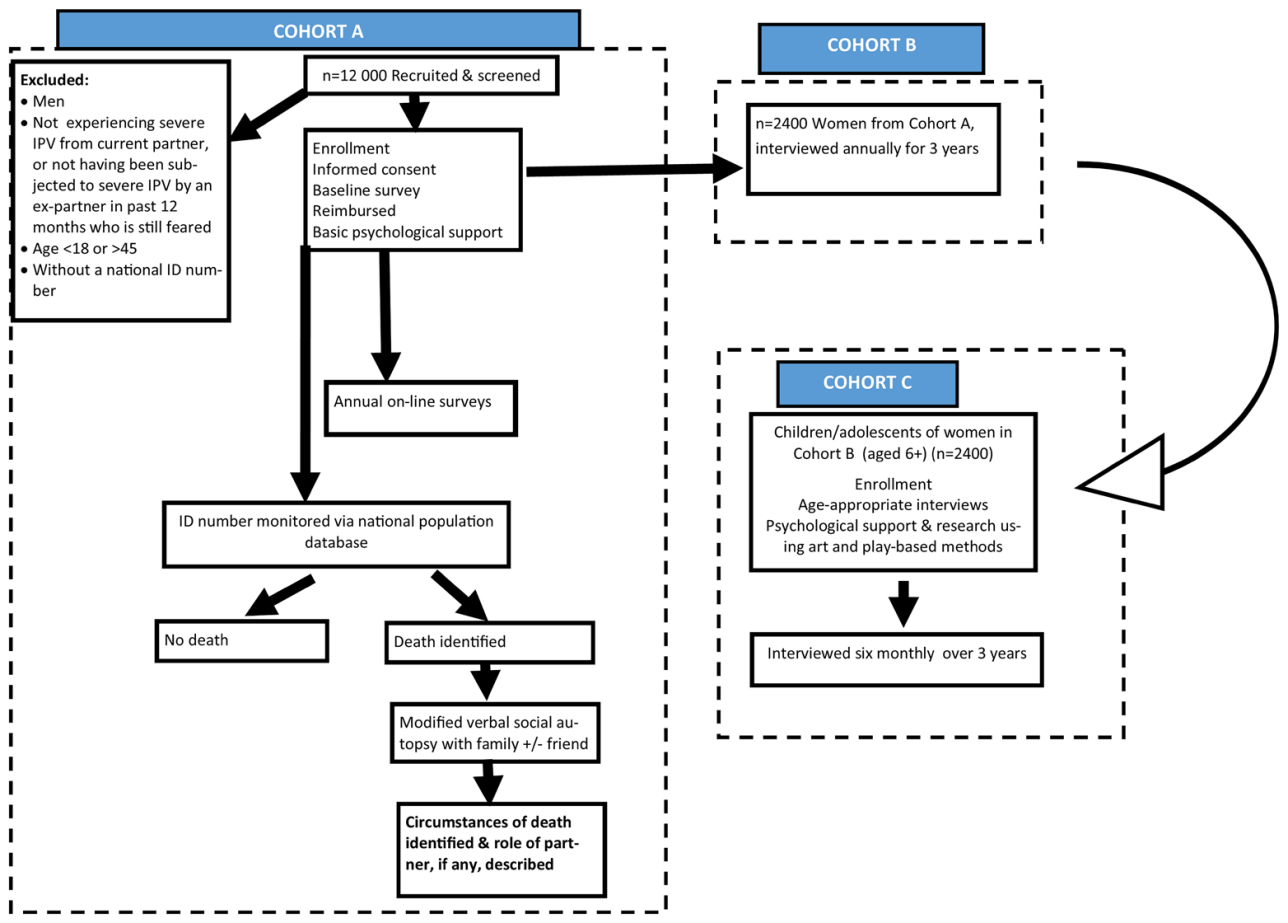
Study procedure for the three cohorts.

The procedure to be followed in the study is outlined in Figure 5. Participants first complete a screening instrument to establish eligibility and record their national identity (ID) number. Then, after the consenting process, they complete the baseline survey, in an interview that generally takes about 2 hours. They sit next to the interviewer with the computer screen between them and co-complete the questionnaire on REDCap. This process greatly enhances the acceptability of the interview experience and enables a fairly long interview to be conducted without participant boredom. All interviews conducted in the office follow this approach. At the end of the survey, they are asked if they can assist with chain referral by taking coupons and asked to give consent for further research. Each participant is offered counselling or other assistance from an on-site social worker. This cohort is currently enrolling participants.

All participants are checked against the National Population Register provided weekly to the South African Medical Research Council by the National Department of Home Affairs. This contains all registered deaths in the country, with fields for national ID number, place of registration, date of death and whether the death was due to ‘natural’ or ‘unnatural’ causes. The ID numbers of all participants in the study are merged and compared to the National Population Register fortnightly to determine if there are deaths. All study participant deaths will be reported to the research ethics committee, and we will use information available in the participant tracking form, as well as information held on the participants’ referral to the study, to identify persons who may have knowledge of the death and conduct a verbal social autopsy. The aim of the verbal autopsy is to establish the probable cause of death and, if the death was unnatural, circumstances of death with particular focus on a possible link to IPV, the suspected perpetrator in the case of a murder, and help-seeking (if any) in the period prior to the death, to determine if there may have been any missed opportunities for femicide prevention. The verbal autopsy will use mixed methods, with a brief structured questionnaire in REDCap and a more extensive qualitative interview. Approaching bereaved family and friends always requires sensitivity, but when this is done, we have found them willing to share information.

After cohort recruitment, all participants will be invited to complete an annual interview with the invitation sent by cellphone to those having their own phone/SIM card. For safety, we will only invite participants for follow up interviews if they have private access to a cellphone. The messages will include a link that can be used to complete the survey that is password protected. The brief annual surveys will capture key time-varying variables including change in relationship status, IPV help-seeking, injuries from violence and hospitalization, depression and well-being.

Participants enrolled in the first sub-cohort (Cohort B) will be invited for a follow-up interview about 6–12 months after the baseline interview. Thereafter they will be invited annually for the following two years. This will provide three post-baseline interviews and, should resources permit, they will be invited for further interviews. The first interview with children will be held after the sub-cohort (Cohort C) interview with their mother. Children will be interviewed 6 monthly over 3 years. Data will be collected from the children in a structured interview, from the mother, reporting on the child, and in some direct measurements of e.g. height and weight. In the interviews with younger children, arts and play-based methods will also be used as components of the data collection. These methods will serve the dual role of assisting discussion about violence and safety at home, fear, and feelings, as well as opening conversations about mother and child relationships. An art and play-based pilot intervention will be developed for children and adolescents, that is intended to provide therapeutic value. Two sessions of the programme will be dyadic, including mothers, to strengthen maternal-child/adolescent relationships and understanding.

## Data analysis

The data are entered directly into REDCap. The database is cleaned regularly and managed by the study statisticians. Data analysis will be conducted in Stata version 17.

We will establish risk factors for IPF and attempted IPF by comparing social, economic and relationship circumstances, childhoods, mental health, partner characteristics and help-seeking of women killed by their current or former intimate partners with those not killed. We will use both cross-sectional and longitudinal statistical modelling techniques.

Several longitudinal statistical modelling techniques will be used to investigate the risk factors for intimate partner femicide. For example, longitudinal structural equation modelling (LSEM) will be used to investigate the relationship between women’s mental health and violence experienced, while controlling for time-varying mediating factors such as help-seeking and other confounders. Other longitudinal statistical methods such as marginal structural models (MSM) will be used to assess causal effects of time-varying exposures (e.g. partner substance abuse) on women’s risk of IPF. Marginal structural models estimate the effect of time varying treatment/exposure on outcome by allowing for appropriate control for the effects of time-varying confounders.

The use of the art and play-based research data will be tested initially in a pilot study and methods thereafter finessed. They will be analysed by coding the discussion around the creative products (e.g. pictures, sand boxes images), using qualitative research methods. The aim will be both to understand what is expressed in the discussions as well as to determine which outcomes of the dyad might be feasibly captured using closed codes to create variables to be included in the REDCap dataset for analysis with the survey data. The discussions around the images will be recorded by note-taking and audio-recorded, and with permission, we will record the final creative products with a photograph. A sub-sample of recordings will be transcribed verbatim and translated into English in preparation for data analysis. We will use thematic analysis to inductively analyse the qualitative data. The audio recordings of interviews from the verbal autopsy will be handled in a similar manner.

## Ethical and safety considerations and participant support

The protocol has been approved by the South African Medical Research Council’s Human Research Ethics Committee (EC032-5/2024, 28 May 2024). This study is being conducted according to the generic ethical principles for research outlined by the Council for International Organisations of Medical Sciences (CIOMS) ^48^. In addition, the research will follow the WHO guidelines Putting Women First: Ethical and Safety Recommendations for Research on Domestic Violence Against Women (2001) ^49^. Where relevant, for research on GBV perpetration, we will follow Ethical and Safety Recommendations for Research on the Perpetration of Sexual Violence ^50^. With respect to research with children, we will follow the recommendations on ethical principles of the report of the Child Protection Monitoring and Evaluation Reference Group of UNICEF developed in response to gaps and challenges in child protection research ^51^.

All research follows the foundational principles of respect for persons and justice. The study will be publicly named ‘Fediša Modikologo: end the cycle’ alternatively the Fediša Women’s Health Study. These names do not mention ‘violence’ and so provide non-stigmatising branding. We will avoid publicly linking the study name to the notion of ‘violence’ until data collection across all cohorts has been completed. All participants are to be interviewed in private in an office providing appropriate auditory and visual privacy, in a rare situation where we may interview a participant at home, such as when conducting verbal autopsies, we will ensure privacy there as well, and our staff are trained to pause the interview should there be an interruption by a third party. We are providing information on the study to all participants and seeking written informed consent prior to participation. All participants receive financial reimbursement for their time and travel. For Cohort C, we will recruit children into the study via their mothers and gain consent for participation from their mothers and assent from the children. All participants will be able to decline further participation at any stage without consequences. Protecting participants from harm or abuse is a major consideration in all our research. To spread the burden of research, we will use the four offices which enable us to access all linguistic and racial groups in the country. We also hope to recruit from across all socio-economic groups. However, we will exclude foreign nationals from the study because they do not have the national identity numbers that we are using to track deaths, and we will exclude some South African national who do not have an ID number. We recognize the risks foreign nationals face, and the vulnerability of nationals without an ID number, and recognize this as a limitation of the study.

Interview quality is vital for the data and for ethical data collection. All staff conducting interviews are trained in sensitive interviewing, in order to identify when pauses and refreshments are needed in the interview, as well as containment and distress management in an interview environment. Recognising that we are recruiting very vulnerable participants, each office has a professional social worker and auxiliary social worker as the staff who counsel participants and assist in complex situations, such as helping women who perceive they have an acute need of protection, link to the police or get Protection Orders, and mandatory referrals to social services, as well as assisting in social grant applications or referral to health services, or shelters. We have developed a distress protocol and standard operating procedures (SOPs) to guide this work. The social workers also manage a discretionary fund, and donations, that can be drawn upon when there is an acute need for food items or vouchers, according to a SOP.

Support for child participants will also be provided by the social workers. In addition, we will offer all children a four-session art and play-based intervention that will enable discussion of risk and safety at home, and help to identify children with special needs. Two sessions will include their mother and we hope these will facilitate relationship strengthening. The intervention will be developed by a clinical psychologist specializing in art and play therapy, and will be designed for use by trained and supervised lay practitioners, including the social work team. Monitoring and evaluation of these sessions will be conducted on an on-going basis, to prevent adverse events.

The study has safety and referral protocols for adults and children. These will include referral of participants with severe depression, PTSD and those who screen positive for suicidality to local professional counselling services. We also undertake mandatory referrals as per South Africa’s Children’s Act (2008) and Domestic Violence Amendment Act (of 2021), and our study social workers are responsible for facilitating this and ensuring interventions are conducted by the legislated authorities. The Children’s Act mandates reporting of any form of child abuse or neglect to social workers, and the Domestic Violence Amendment Act also mandates reporting of children’s exposure to domestic violence, in some circumstances, to the police. We are handling this sensitively, working with the study social workers, and have designed the research to avoid collecting certain types of information that may be reportable. When reports are made, we will manage referrals in a way that builds relationships with the children’s mother, where possible, even when it may be perceived that an act or omission on her part has precipitated the referral.

All staff working on the study have ethics training, as evidenced by a valid Good Clinical Practice (GCP) certificate and have attended personal development training provided by the non-governmental organisation, Lifeline, which enables them to process some of their emotions and life experiences from prior to the study. In addition, staff are trained on ethical and safety considerations in conducting research on VAW, as well as the specific training related to the study protocols. In order to reduce the risk of vicarious trauma experienced by staff, interviews are conducted no more than four days per week, and informal daily debriefing is encouraged, as well as weekly group debriefing within the team and quarterly group debriefing with a psychologist. Individual staff counselling can be accessed when needed via the SAMRC’s Employee Wellness Programme.

All data collected in the study are kept confidential in password and firewall protected databases, as per the SAMRC data protection systems. We are collecting and retaining personal participant information to use for cohort tracking and retention purposes, including information on friends and/or family. This we keep securely, separate from the questionnaire database. Further we have recruited participants through chain referrals, the details of the participant initiating this recruitment are also retained. In the event of a death, this tracking information will be used for us to ascertain the circumstances of death and complete the verbal social autopsy. In cases of suspected homicide, we will engage with the police if it seems to be appropriate, and, if interviewees give consent, we may share any information with the police that may be needed, and of assistance, in the case investigation. This could be seen as a possible breach of confidentiality, however, we will be guided by our belief that our participants deserve justice after a violent death and that their best interests are served by assisting the justice system, if this is needed.

## Stakeholder involvement and women with lived experience groups

Stakeholders are involved in several ways, through networking with key service providers, government departments and NGOs that provide support and services for IPV survivors, in each of the four sites and nationally. Furthermore, Community Advisory Boards (CABs) were established at each site and nationally. The CABs have approximately 15 members who provide advice and insight on the research questions, methods, recruitment and will assist with research uptake once results become available. Membership includes women with lived experience of severe IPV, service providers (government and community), NGOs working on reducing IPV, government officials and researchers. They meet four – six monthly. CAB members are provided lunch, but not compensated, except for lived experience representatives who are given ZAR200 (approx. US$10) plus transport per meeting.

There is growing recognition of the importance of involving people with lived experience in research. Engaging women with lived experience (WLE) enhances the understanding of the research team, helps with disseminating findings to appropriate audiences, and is empowering for the women themselves ^52^. WLE are recognised as ‘experts by experience’, and they assist in identifying the key research questions for Cohort B, understanding community needs, interpreting findings, participant recruitment, and assist the project team to reflect upon how we respond to challenges experienced throughout the study. Recruitment to WLE groups is complicated by the need for safety. Most participants are still in violent relationships and so there can be risks associated with WLE group involvement. To navigate these, each WLE group comprises of 7–12 women from the communities who have left severely violent intimate relationships, or have chosen to remain with a partner who has used violence, but is not severely controlling. Some members are WLE activists, and despite being ineligible for the study due to being above maximum recruitment age or having been free of their partner for more than 12 months, they are included and make a valuable contribution. Each site has a WLE group that meets separately from the CAB, prior to CAB meetings, and members are compensated and receive travel reimbursement and lunch. Two members of the WLE attend the CAB and we try to ensure that their meetings precede the CAB and they are introduced to the presentations for the CAB prior to the meetings so that they can reflect on these in a safe space.

## Strengths and limitations

The strengths of this study include its highly experienced VAW research team, which draws on the disciplines of epidemiology, psychology, biostatistics, social sciences, and health economics, and has research and practitioner partners. It has been established as longitudinal research and has been funded to collect data over sufficient years to be able to substantially advance knowledge. The research sites are rooted in the local communities in which they are based with active community advisory boards and lived experience groups. Furthermore, the study has been developed after close consultations with Government Departments, particularly the Department of Justice and Constitutional Development, and intends to answer some of the key research questions for these Departments. This is the first time that a study of this nature has been attempted in any setting, and it is an unfortunate feature of South African society, with the country’s very high prevalence of femicide and severe IPV, that the study is even possible with the present design.

This study has a number of limitations. We recognize that severe IPV and IPF affect women from all social circumstances and ages in South Africa and that many women experiencing severe IPV are not networked to others with similar experiences or using services, and thus there are some women who cannot be accessed through our methods. We are also aware that some women experiencing severe controlling behaviour are unable to contact our sites for interviews, do not live near one, or do not feel sufficiently safe to do so. This study is not focusing on severe IPV in same-sex relationships, however it is possible for IPF by a female/non-binary intimate partner to be identified and described through the verbal autopsies. As a study based on cohorts, we are not able to calculate population prevalence and may have unrecognised biases in the sample. Further, we recognise that women who do not hold South African ID cards are at risk of severe IPV and IPF and they are not included in our study. However, we do not expect that the omission of some sections of the population experiencing severe IPV will detract from the validity of the main results.

Our sample size calculation has been based on a series of assumptions about the prevalence of IPF and attempted IPF in our cohort, however, the annual incidence and prevalence of these among women experiencing severe IPV in South Africa is not known. Thus, the study maybe under-powered in relation to IPF. Among the first study participants, approximately two-thirds have ever experienced attempted IPF and so we anticipate having little difficulty in being sufficiently powered in respect of attempted IPF.

The study has multiple elements that are longitudinal, including the ascertainment of deceased participants, the annual self-completed interviews, and the nested sub-cohorts, and we recognize that there can be error in conclusions drawn from longitudinal analyses of data where follow-up is incomplete. We anticipate being able to identify at least 90% of women who die through the National Population Register and we expect to be able to find details of the deaths in most cases through the verbal autopsy. This will be a substantial improvement on the routinely available data ^53^. There may be some bias in any cases that we cannot follow up as they may be more socially isolated. Unfortunately, we do not have the resources for active follow up of the main cohort and so the 20% that we will be able to actively follow up will feature disproportionately in all further analyses, as well as the sub-group of women who do respond positively to invitations to complete the on-line annual survey. Whilst women who can respond to the on-line survey will differ from those who cannot, particularly socio-economically, we will try to redress this bias in out sub-cohort recruitment. We hope the fact that we have completed the main cohort interview prior to inviting participants to join the women’s sub-sample cohort, will enhance retention in that cohort, and in turn the mothers’ participation will enhance retention of the children. All sensitive surveys of the type used in this study have a risk of under-disclosure of aspects of a participant’s personal life. We hope that the rigorous training of our staff and the conducive environment of our offices will provide a context for interviews that is most likely to enable free disclosure.

Previous research on IPV prevention has found that studying IPV in a population, even without any intervention (as in no-intervention control arms), can result in a reduction in reported IPV prevalence over time ^54^. We recognise that recruitment into our study, with the interviews and consultation with a social worker, may in itself impact women’s lives and the violence they experience. Indeed, some participants have mentioned that their interview helped them. This will introduce some bias into the results, with the likely result being that we would need a larger sample size to detect smaller effect sizes. However, if our work enhances the protection for our study participants and prevents mortality we will consider the statistical impact a small price to pay.

## Dissemination

The findings of the research will be published in academic peer-reviewed journals and presented at conferences, and key findings will be presented in summary format for a practitioner and policy-maker audience in South Africa. We will share emerging findings with the CABs and WLE groups at their meetings and seek their advice on interpretation and dissemination of findings. In the locality of each study site, we have connected with the local community on multiple levels, and we will feed our findings back at intervals. We will also hold seminars with practitioners and policymakers to discuss the interpretation of findings and their implications for policy and practice, and disseminate through the news and social media.

## Conclusions

This research seeks to answer key research questions around risk factors for IPF and the impact of severe IPV on women and their families, and the benefit of assistance that is provided through our laws and services. It will provide unparalleled insights into the risk that South African women face in relationships, and the impact of violence on their children and adolescents, and advance our understanding of the phenomenon of intergenerational cycling of violence. These will provide vital foundations for developing more effective approaches to IPF prevention, as well as deepening our understanding of how to assist women, facing severe IPV, to free themselves from violence, and what is needed to effectively assist their children and adolescents. These findings will be of considerable benefit to practitioners, policymakers and researchers working to end the scourge of VAW and violence against children.

## Ethics and consent

The protocol has been approved by the South African Medical Research Council’s Human Research Ethics Committee (EC032-5/2024, 28 May 2024). The pilot studies were given approval by the same committee (EC032-9/2023) and (EC032-11/2023). Fediša is being conducted according to the generic ethical principles for research outlined by the Council for International Organisations of Medical Sciences (CIOMS) ^48^. In addition, the research will follow the WHO guidelines Putting Women First: Ethical and Safety Recommendations for Research on Domestic Violence Against Women (2001) ^49^. Where relevant, for research on GBV perpetration, we will follow Ethical and Safety Recommendations for Research on the Perpetration of Sexual Violence ^50^. With respect to research with children, we will follow the recommendations on ethical principles of the report of the Child Protection Monitoring and Evaluation Reference Group of UNICEF developed in response to gaps and challenges in child protection research ^51^.

All participants sign informed consent for interviews and maternal consent will be sought for child participants, with assent from children.

## Data Availability

No data are associated with this article.
