# Supplementary material for: Fediša Modikologo: breaking the intergenerational cycle of violence against women and children. Theoretical framework and protocol for a prospective cohort study
Source: Wellcome Open Res. 2025 Aug 7;10:126. Originally published 2025 Mar 7. [Version 2] doi: 10.12688/wellcomeopenres.23513.2 (PMC12287687; doi:10.12688/wellcomeopenres.23513.2)
Supplement: Peer review history

# Reviewer report: Reviewer response for version 2

**DOI:** 10.21956/wellcomeopenres.27306.r128661

**Published:** 2025-08-27

**Authors:** Janina Isabel Steinert

**Affiliations:** 1 Technical University of Munich, Munich, Germany

The protocol is excellent and the edits also clarify all remaining questions I had. I wish to commend the authors for such a strong research design and am looking forward to seeing the empirical findings of this study in the future.

Is the study design appropriate for the research question?

Yes

Is the rationale for, and objectives of, the study clearly described?

Yes

Are sufficient details of the methods provided to allow replication by others?

Yes

Are the datasets clearly presented in a useable and accessible format?

Not applicable

Reviewer Expertise:

Gender-based violence

I confirm that I have read this submission and believe that I have an appropriate level of expertise to confirm that it is of an acceptable scientific standard.

# Reviewer report: Reviewer response for version 1

**DOI:** 10.21956/wellcomeopenres.25934.r125247

**Published:** 2025-07-23

**Authors:** Md. Mahfuz Al Mamun

**Affiliations:** 1 Maternal and Child Health Division, icddr, Dhaka, Bangladesh

This is an excellent protocol with clear rationale and objectives of the study. The study design has been clearly described and appropriate to answer  the research questions. The theoretical frameworks of the study are easily understandable. Participants ad participant recruitment process have been clearly described. Sample size looks appropriate to achieve the power and the description of the power calculation in the paper is excellent. Measurement section is very detailed and clear and with sufficient citations. Procedure has been written in details. Ethical and safety considerations and participant support have been clearly mentioned. Stakeholder involvement and Women with Lived Experience Groups is a great strength of this research. Finally, sufficient details of the methods provided to allow replication by others.

Is the study design appropriate for the research question?

Yes

Is the rationale for, and objectives of, the study clearly described?

Yes

Are sufficient details of the methods provided to allow replication by others?

Yes

Are the datasets clearly presented in a useable and accessible format?

Not applicable

Reviewer Expertise:

My areas of research include gender, violence against women and girls (VAWG), violence against children (VAC), intergenerational transmission of violence, impact of violence on mental health, child marriage, sexual and reproductive health and rights (SRHR)

I confirm that I have read this submission and believe that I have an appropriate level of expertise to confirm that it is of an acceptable scientific standard.

# Reviewer report: Reviewer response for version 1

**DOI:** 10.21956/wellcomeopenres.25934.r125246

**Published:** 2025-07-14

**Authors:** Janina Isabel Steinert

**Affiliations:** 1 Technical University of Munich, Munich, Germany

The protocol describes an excellent and very well-designed study that aims at generating much needed evidence on an under-researched but crucial topic: intimate partner femicide in South Africa. The longitudinal design and rich questionnaire will allow for an in-depth investigation of factors associated with intimate partner femicide and help identify important target points for prevention efforts. Particular strengths include the involvement of women with lived experiences throughout the stages of the research project and the very thorough strategies outlined to ensure the ethical integrity of the project and the safety and wellbeing of all involved, both study participants and research staff.

 I would first like to share some broader comments on the study design and methodology:

 1. Could the authors provide some justifications for their choice of severity indicators, which they use to distinguish "severe IPV" from other IPV? How were these indicators for the different subforms of IPV selected and why were they chosen over others? For example, why was women's inability to earn own income included but partners stealing women's income was not? Or why was stalking, for example, included but emotional/psychological abuse in the form of degrading or insulting a partner was not?  Also, there needs to be additional clarity on this: "Alternatively, she would have experienced multiple forms of severe controlling behaviour, such as being confined to the home, prevented from working or earning income, or experienced jealous surveillance." - What means having experienced "multiple forms"? How many of these? How was the cut-off defined? 

 2. I was a bit confused about the inclusion of tech-facilitated IPV (see Figure 1).  There was no explanation of this measure in the text itself and I would need to see some examples of how the authors intend to measure tech-facilitated violence specifically perpetrated by the partner since I assume most tech-facilitated IPV like sending of dickpicks etc. is perpetrated by strangers and anonymous social media users. Does this measure really fit here? Was it at all included in the definition of severe IPV? I assume not?

 3. In terms of risk factors, I would suggest differentiating between internalised gender norms/attitudes (i.e. what women themselves believe) and perceived community social norms (i.e. what they think people in their surroundings believe). This could help address and identify the possible issue of pluralistic ignorance.

 4. "In order to achieve the second aim, a sub-cohort of women from the cohort A will be followed intensively to study impact of severe IPV on their and their families’ lives (Cohort B)." - I did not fully understand how the sub-cohort B will be selected? Will this only include women who have been flagged for severe IPV in cohort 1? How many women of Cohort A are to be included in Cohort B? There could be some more clarity on this process.

 5. The presented power calculations should also include some information on the estimated retention rates across the waves. Can the authors draw on existing longitudinal studies in the same or a similar context to generate a lowerr- and upper-bound estimate of this rate? 

 6. There needs to be more information on the interview mode for the different cohorts and for the different survey waves. It seems that the authors will use a mix between face-to-face and online surveys? But this was not sufficiently clear and needs to be specified in more detail. If the authors use face-to-face interviewing, will they make use of ACASI technology?

 7. Overall timeline: I was a bit disappointed to see that the longitudinal data will only cover a period of 2 years, especially given the (let's hope so!) rarity of the main outcome. How do the authors see the following suggestion: Could the overall time period be extended to 4,5 years with only one interview per year? I feel that this could allow for more insightful data overall and possibly capture some longer-term time trends.

 8. "The use of the art and play-based research data will be tested initially in a pilot study and methods thereafter finessed. They will be analysed by coding the discussion around the creative products (e.g. pictures, sand boxes images), using qualitative research methods. " - I did not fully understand whether this qualitative data collection only concerns the pilot activities or whether this will be an integral part of the main longitudinal study. 

 Minor comments:

 1. The introduction section includes some typos/grammar issues, and I recommend a thorough proofread. Listing some of the issues I spotted below:

 - "Intimate partner femicide (IPF) is the most extreme consequence of intimate partner violence (IPV), and usually precedes the fatal event" - "and" needs to be replaced with "which".

 - "... IPF surveillance has been conducted in through dedicated surveys..." - cross out the "in"

 - "The countries reporting the highest rates being Surinam (4.3/100,000 female population) and Belize (2.1/100,000 female population) ^4^." - change "being" to "are".

 -"but there is also a story about the loyalty of many women who experience IPV towards their abuser, and the pressures they may experience to stay, or leave, these relationships from family and communities" - "there is a story about..." sounds colloquial - suggest rephrasing

 2. Could the authors provide some context as per the study's name? What does  Fediša mean? Why was this name used?

 3. Theoretical framework for the study: "The aims will be researched through a study with three nested cohorts, and associated research." - Here, it was not yet clear what the authors mean by "nested cohorts". Could they add one sentence in here to clarify this?

 4. While I appreciate Table 3, I would prefer to see a more detailed table with all specific items included for each of the constructs in the appendix.

 5. One additional limitation to add is that the sample is not representative of the general population. The data is therefore not suitable for establishing the "true" prevalence of general IPV and may over-estimate this rate. This being said, I still believe the sample is very suitable for estimating the prevalence of intimate partner femicide.

Is the study design appropriate for the research question?

Yes

Is the rationale for, and objectives of, the study clearly described?

Yes

Are sufficient details of the methods provided to allow replication by others?

Yes

Are the datasets clearly presented in a useable and accessible format?

Not applicable

Reviewer Expertise:

Gender-based violence

I confirm that I have read this submission and believe that I have an appropriate level of expertise to confirm that it is of an acceptable scientific standard, however I have significant reservations, as outlined above.

# Reviewer report: Reviewer response for version 1

**DOI:** 10.21956/wellcomeopenres.25934.r120439

**Published:** 2025-03-27

**Authors:** Reshmi Mukerji

**Affiliations:** 1 Independent Consulant, Kolkata, India

The article proposes a longitudinal cohort study to understand the risk factors for intimate partner femicide,  the impacts of severe IPV on women and their families, and the risks and drivers of intergenerational cycling of violence. It also aims to describe how help seeking and community resources aids in recovery and protects from further violence. 

 I only have some minor comments to add:

 1. Theoretical framework: "Her partner’s risk factor": Should it also include partner's exposure to/witnessing of IPV as a child?

 2. Table 3: (i) For the "relationship with mother" variable please correct typo "behaviour" should be changed to "behaving"

 3. Inclusion and exclusion criteria: While inclusion criteria has been described in detail on pages 5-6, the exclusion criteria can only be seen in Figure 5 and later "the exclusion of foreigners" described in page 15. Please add foreigners/those without national ID number to figure 5. In addition, would suggest adding a description of the full exclusion criteria in the text possibly following the paragraph where inclusion criteria has been described.

 4. Selection into Cohort B: A little bit of additional detail will be helpful. How is selection done for entry into Cohort B, ie, is everyone who is asked and agrees to participate in the cohort B included or is there a selection criteria? What if more women than the 20% needed agree to participate in the Cohort B, how will they be excluded?

 5. Data collection with children (Art and play): From what I understand, the play sessions with the children will also be where data is collected for the children’s interviews? Is this correct? Will mother and children's dyadic data be collected from these sessions or just children's? A couple of sentences to clarify in the data analysis section will be helpful.

 6. Remit of IPF/attempted IPF: Will suicides (recorded in the death registry or from verbal autopsies) or attempted suicides be considered within the remit of IPF given that men’s behaviours may have abetted the suicide? In the Indian setting it has been seen that many housewives "suicides" have actually been a direct result of IPV from partners and in-laws. Can the same be possible in the South African setting?

 7. Verbal autopsies: Considering the sensitivity of the topic, how will verbal autopsies will be conducted with friends and family of the deceased (eg recruitment, site of interviews, number of people interviewed, individual vs group interviews)? How will the safety of these participants be ensured given that the perpetrator might still be living in the community? Adding a bit more detail will help clarify these ethical issues. 

 8. Police reporting: On page 15-16 it is mentioned that information might be shared with the police in cases of homicide. In such cases how will the issue of consent be established/breach of confidentiality issues be handled?

 9. WLE and CAB groups: Adding a bit more clarity about how WLE groups will be separate from the CAB and the unique contributions of each will be helpful.

 10. Qualitative research: It seems qualitative research for the study was restricted (i) to cognitive testing of measures, and bette understanding of contracts to improve the surveys, (ii) analysis of art of play sessions with children, (iii) verbal social autopsy data. Giving the layered complexity of the topic of study, I was curious why a qualitative component with survivors of severe IPV was excluded from the study design? Would it not be helpful to conduct qualitative interviews with a sample of women from Cohort B to understand the complexities of interconnected social issues such as stigma, societal expectations of leaving/staying with abusive partners, structural difficulties, mental distress, etc that IPV survivors have to navigate and that also might contribute to the intergenerational cycle of violence?

Is the study design appropriate for the research question?

Yes

Is the rationale for, and objectives of, the study clearly described?

Yes

Are sufficient details of the methods provided to allow replication by others?

Yes

Are the datasets clearly presented in a useable and accessible format?

Not applicable

Reviewer Expertise:

(1) Qualitative methodology, (2) VAW, (3) Stigma

I confirm that I have read this submission and believe that I have an appropriate level of expertise to confirm that it is of an acceptable scientific standard.

# Response

**Published:** 2025-08-06

**Authors:** Rachel Jewkes

**Affiliations:** Office of the Executive Scientist, South African Medical Research Council, Pretoria, Gauteng, South Africa

The article proposes a longitudinal cohort study to understand the risk factors for intimate partner femicide, the impacts of severe IPV on women and their families, and the risks and drivers of intergenerational cycling of violence. It also aims to describe how help seeking and community resources aids in recovery and protects from further violence. I only have some minor comments to add:

 1. Theoretical framework: "Her partner’s risk factor": Should it also include partner's exposure to/witnessing of IPV as a child?

**Authors’ response** We totally agree and realise its not very clear from what we have written. We have edited the figure and the text. Below is the revised paragraph: *The aims will be researched through a study with three nested cohorts, and associated other research. Figure 1 presents the theoretical framework for the main cohort in this study (Cohort A). We hypothesise that a woman’s risk of femicide, or attempted femicide, is a product of the violent and controlling practices that she experiences from her male partner in the relationship. It will be further influenced by a range of forces that stem from her background and socio-economic circumstances, influenced by her experiences of trauma, violence and key relationships in her childhood, including witnessing abuse of women at home. Her mental and physical health, resilience and coping, including substance misuse, will be influenced by her experiences of violence, and these will also impact her response in the face of further IPV. Her risk will also be influenced by her partner’s social and economic circumstances, the degree to which he embraced ideals of hegemonic masculinity with the associated entitlements, peer influences, and norms related to the use of violence, as well as by his personality, mental health and substance use. These will also be shaped by his exposure to violence in childhood and witnessing abuse. We hypothesise that how she responds, for example by leaving or staying, will influence her subsequent IPV exposure. Her continuing risk of IPV will also be related to her experiences in help-seeking for relationship problems and the internal and external pressure that she encounters to stay or leave the relationship, and whether she has children. As we are not interviewing her partner, we are only able to collect such information as we can through her reports. This limits our ability to understand more about his background and childhood and we have found most women do not know very much.*  

 2. Table 3: (i) For the "relationship with mother" variable please correct typo "behaviour" should be changed to "behaving"

**Authors’ response** Thanks we have correct this.

 3. Inclusion and exclusion criteria: While inclusion criteria has been described in detail on pages 5- 6, the exclusion criteria can only be seen in Figure 5 and later "the exclusion of foreigners" described in page 15. Please add foreigners/those without national ID number to figure 5. In addition, would suggest adding a description of the full exclusion criteria in the text possibly following the paragraph where inclusion criteria has been described.

**Authors’ response** Thanks, we have edited the figure. The eligibility criteria are outlined in the section labelled ‘Participants’ on page 11 of the file. We have assumed that that reader will recognise that if these are the eligibility criteria, that a person will be ineligible if they do not meet the criteria.

 4. Selection into Cohort B: A little bit of additional detail will be helpful. How is selection done for entry into Cohort B, i.e., is everyone who is asked and agrees to participate in the cohort B included or is there a selection criteria? What if more women than the 20% needed agree to participate in the Cohort B, how will they be excluded?

**Authors’ response** We have given a description on manuscript age 12 and added the following to clarify that we will choose among the women and not take everyone. *Much of our decision-making will be driven by the need to meet these criteria, but where we have choice due to a larger number of potential participants than required, we will randomly select among them.*

 5. Data collection with children (Art and play): From what I understand, the play sessions with the children will also be where data is collected for the children’s interviews? Is this correct? Will mother and children's dyadic data be collected from these sessions or just children's? A couple of sentences to clarify in the data analysis section will be helpful.

**Authors’ response** We are going to collect some data in play sessions with younger children, but the main data collection will be via a formal interview. We will collect data from the mother about the child as well, but none of the interviews will be in the presence of the other person.  We have edited the main paragraph describing Cohort C as follows. We will publish a separate methods paper describing this, when we have completed formative research, pilot testing and finalised all the methods to be used. *Participants enrolled in the first sub-cohort (Cohort B) will be invited for a follow-up interview about 6–12 months after the baseline interview. Thereafter they will be invited annually for the following two years. This will provide three post-baseline interviews and, should resources permit, they will be invited for further interviews. The first interview with children will be held after the sub-cohort (Cohort C) interview with their mother. Children will be interviewed 6 monthly over 3 years. Data will be collected from the children in a structured interview, from the mother, reporting on the child, and in some direct measurements of e.g. height and weight. In the interviews with younger children, arts and play-based methods will also be used as components of the data collection. These methods will serve the dual role of assisting discussion about violence and safety at home, fear, and feelings, as well as opening conversations about mother and child relationships. An art and play-based pilot intervention will be developed for children and adolescents, starting with the first interview, that is intended to provide therapeutic value, as well as enabling data collection on safety and violence exposure. Two sessions of the programme will be dyadic, including mothers, to strengthen maternal-child/adolescent relationships and understanding.*

 6. Remit of IPF/attempted IPF: Will suicides (recorded in the death registry or from verbal autopsies) or attempted suicides be considered within the remit of IPF given that men’s behaviours may have abetted the suicide? In the Indian setting it has been seen that many housewives "suicides" have actually been a direct result of IPV from partners and in-laws. Can the same be possible in the South African setting?

**Authors’ response** In South Africa, we do not include suicide in our definition of intimate femicide. We agree men’s behaviours may have abetted suicide, as they also abet deaths from other causes, including HIV. We are concerned that a death classified as ‘suicide’ may not be recognised as having been caused by a man’s behaviour and we hope to shed light on that through our use of verbal autopsy.

 7. Verbal autopsies: Considering the sensitivity of the topic, how will verbal autopsies will be conducted with friends and family of the deceased (eg recruitment, site of interviews, number of people interviewed, individual vs group interviews)? How will the safety of these participants be ensured given that the perpetrator might still be living in the community? Adding a bit more detail will help clarify these ethical issues.

**Authors’ response** Approaching bereaved family and friends always requires sensitivity, but we have ways of doing it and have found them willing to share. It may be that in South Africa, cause of death may not be quite as sensitive as in other settings as obituaries frequently state it and it is open spoken of at funerals. We don’t particularly anticipate security issues related to doing the verbal autopsy. We have added the following sentence to the end of the paragraph on p.31-32. *Approaching bereaved family and friends always requires sensitivity, but when this is done, we have found them willing to share information.*

 8. Police reporting: On page 15-16 it is mentioned that information might be shared with the police in cases of homicide. In such cases how will the issue of consent be established/breach of confidentiality issues be handled?

**Authors’ response** We have a consent form for relatives to complete if they agree to this.  We have lightly edited the section so it now reads: *In cases of suspected homicide, we will engage with the police if it seems to be appropriate, and, if interviewees give consent, we may share any information with the police that may be needed, and of assistance, in the case investigation. This could be seen as a possible breach of confidentiality, however, we will be guided by our belief that our participants deserve justice after a violent death and that their best interests are served by assisting the justice system, if this is needed.* We have thus far only had one femicide, and we didn’t share any information, or particularly get involved, because the police had made an arrest and had a confession, we were told. So it will depend on the circumstances.

 9. WLE and CAB groups: Adding a bit more clarity about how WLE groups will be separate from the CAB and the unique contributions of each will be helpful.

**Authors’ response** We are planning a separate paper describing the WLE groups. We have extended the account as follows: *Each site has a WLE group that meets separately from the CAB, prior to CAB meetings, and members are compensated and receive travel reimbursement and lunch. Two members of the WLE attend the CAB and we try to ensure that their meetings precede the CAB and they are introduced to the presentations for the CAB prior to the meetings so that they can reflect on these in a safe space.*

 10. Qualitative research: It seems qualitative research for the study was restricted (i) to cognitive testing of measures, and better understanding of constructs to improve the surveys, (ii) analysis of art of play sessions with children, (iii) verbal social autopsy data. Given the layered complexity of the topic of study, I was curious why a qualitative component with survivors of severe IPV was excluded from the study design? Would it not be helpful to conduct qualitative interviews with a sample of women from Cohort B to understand the complexities of interconnected social issues such as stigma, societal expectations of leaving/staying with abusive partners, structural difficulties, mental distress, etc that IPV survivors have to navigate and that also might contribute to the intergenerational cycle of violence?

**Authors’ response** We will be conducting qualitative research on a range of different issues, but we have not developed proposals for this work in other areas than those listed. We also have a range of qualitative data collected alongside the questionnaire in open questions and a comment log and have started to publish with some of these findings. We have added a new section which reads as follows: Other qualitative research The study has several different types of qualitative data. There are data from the measures development phase of Cohort A, which included discussion of certain issues, with probing and cognitive testing of the questionnaire, which also entailed some broader discussion of the topics. The same approach is planned for Cohort C and will generate similarly rich data. We also have a open-ended questions embedded in the REDCap questionnaire, and a comment log in REDCap which enables comments with further information to be added after any of the questions. This has provided an additional dimension to understanding women’s experiences that normally would not be available. In addition to this we are planning a range of nested qualitative research projects. These are being planned with interviews to be conducted with women experiencing severe IPV and service providers, NGOs and social workers, providing assistance to them.
